# Communicating science in the COVID-19 news in the UK during Omicron waves: exploring representations of nature of science with epistemic network analysis

**DOI:** 10.1057/s41599-023-01771-2

**Published:** 2023-06-05

**Authors:** Kason Ka Ching Cheung, Ho-Yin Chan, Sibel Erduran

**Affiliations:** 1 Department of Education, University of Oxford, 15 Norham Gardens, Oxford, OX2 6PY UK; 2 School of Geography and the Environment, University of Oxford, South Parks Road, Oxford, OX1 3QY UK

**Keywords:** Cultural and media studies, Science, technology and society

## Abstract

News media plays a vital role in communicating scientific evidence to the public during the COVID-19 pandemic. Such communication is important for convincing the public to follow social distancing guidelines and to respond to health campaigns such as vaccination programmes. However, newspapers were criticised that they focus on the socio-political perspective of science, without explaining the nature of scientific works behind the government’s decisions. This paper examines the connections of the nature of science categories in the COVID-19 era by four local newspapers in the United Kingdom between November 2021 to February 2022. *Nature of science* refers to different aspects of how science works such as aims, values, methods and social institutions of science. Considering the news media may mediate public information and perception of scientific stories, it is relevant to ask how the various British newspapers covered aspects of science during the pandemic. In the period explored, Omicron variant was initially a variant of concern, and an increasing number of scientific evidence showed that the less severity of this variant might move the country from pandemic to endemic. We explored how news articles communicate public health information by addressing how science works during the period when Omicron variants surge. A novel discourse analysis approach, epistemic network analysis is used to characterise the frequency of connections of categories of the nature of science. The connection between political factors and the professional activities of scientists, as well as that with scientific practices are more apparent in left-populated and centralist outlets than in right-populated news outlets. Among four news outlets across the political spectrum, a left-populated newspaper, the Guardian, is not consistent in representing relations of different aspects of the nature of scientific works across different stages of the public health crisis. Inconsistency of addressing aspects of scientific works and a downplay of the cognitive-epistemic nature of scientific works likely lead to failure in trust and consumption of scientific knowledge by the public in the healthcare crisis.

## Introduction

In the global health pandemic, news media plays a central role in science and risk and communication (Basch et al., 2020; Chan et al., 2021; Evans, 2021; Klemm et al., 2014; Pan and Meng, 2016). Health officials and the government initiate campaigns to tackle the crisis, while news media assess, filter and frame scientific information of campaigns, for instance, a vaccination programme introducing risk and effects to the public (Laing, 2011; Puri et al., 2020). The public receives health information from different sources, interprets differently based on their knowledge and trust in the informants, and subsequently takes action in response to these campaigns (Basch et al., 2020; Laing, 2011). Therefore, news media serve as a communication medium among health professionals, government officials and the public. However, poor communication of science and a lack of trust in news media would result in a failure in the promotion of collective public responses to the pandemic (Hoffman and Justicz, 2016). Availability of balanced and impartial news reports reflects diverse opinions on controversial issues during the COVID-19 pandemic (Stanyer, 2021), which is essential for citizens to trust representations of science in news media as an unbiased dissemination platform.

When news media report health crisis news, especially that of global-scale like COVID-19, they should not only strive for politicising science and should uphold objectivity (Maras, 2013). The frequency of appearance of politicians and scientists should be balanced, in order to avoid attitude polarisation in news reports (Hart et al., 2020; Motta et al., 2020). Such polarisation might affect individuals’ decision-making on health issues, hence creating burdens on the healthcare system and affecting the health of healthcare professionals (Klemm et al., 2014). Therefore, news media should be balanced in representing science as a cognitive-epistemic system as well as a social-institutional system (Erduran and Dagher, 2014). One of the current strands of research is framing (Nisbet, 2009), which refers to “central organising idea or storyline that provides meaning to an unfolding strip of events” (Gamson and Modigliani, 1989, p. 143). This strand of research analyses the partiality of pandemic news in a period. For instance, Evans (2021) used framing to trace the coverage and representation of the COVID-19 pandemic in two Eswatini newspapers. His study showed that the Eswatini news represented COVID-19 as a distanciated form of the issue at the beginning and later framed it into a localised issue. On the other hand, scholars, meanwhile, are interested in how news articles unbiasedly and accurately report the scientific truth (Hoffman and Justicz, 2016). Regarding the recent pandemic, Mach et al. (2021) analysed COVID-news in newspapers with a range of political orientations in Canada, US and UK. As reflected on the five indicators proposed by Oxman et al. (1993), namely applicability, opinion versus facts, validity, precision, context and global assessment, the right-populated newspapers had a lower scientific quality than left-populated newspapers in their COVID-19 news. However, the abovementioned indicators or theoretical tools do not provide a unified framework to analyse the dynamic science content that balancing cognitive-epistemic and social-institutional aspects.

Moreover, the theoretical tools in science communication during the pandemic crisis by news media did not consider public understanding and engagement in the processes of science (Secko et al., 2013). Scholars have argued that constructive public science communication needs to move from “filling into the knowledge gaps” of laypersons to sharing the processes of science and different forms of scientific knowledge with the public (Erduran, 2020; Reincke et al., 2020). It is vital that scientific reporting of pandemics impartially communicate the nature of evidence and socio-institutional forms of scientific knowledge to the public (Garcia-Carmona, 2021; Mach et al., 2021). Transparent communication of scientific processes that derive scientific knowledge helps the public to assess source credibility (Ngai et al., 2022), hence affects individuals’ behaviour in mitigating healthcare risks, including abiding by social distancing policies (Chan et al., 2023). To understand how representation of the nature of science in news media, we adopt a framework from science education created by Erduran and Dagher (2014) to analyse content in COVID-19 news. Their framework differentiates science as a cognitive-epistemic system and science as a social-institutional system. Such analysis informs communication strategies of news media on how to unbiasedly report the nature of scientific evidence to maintain public trust in the government’s guidance and public responses to the healthcare system (Hoffman and Justicz, 2016; Laing, 2011).

This paper systematically investigates the connections between categories of nature of science in newspapers with a range of political stances (i.e., left and right wings) in the United Kingdom from November 2021 to February 2022. The aim of the empirical study was to better understand how the news media in the United Kingdom engaged with the Covid-19 related information, particularly with respect to how media from different political orientations reflected the nature of science. For each news article, content analysis was carried out on the representations of the nature of science. Nature of science refers to the characteristics of science in a broader sociocultural context (Erduran and Dagher, 2014). Majority of these studies (Basch et al., 2020; Motta et al., 2020) focus on the communication of exacerbation of the spread of disease during the early stage of the COVID-19 pandemic. There is a lack of study that characterises the dynamic and emergent discourse of the nature of science when a country posits itself to the stage of transition from pandemic to endemic. When the world is being considered to enter the endemic phase by some countries, professional guidance has been changing in order to cope with the prolonged healthcare impacts of the endemic (Cook et al., 2021; Hunter, 2020). Although there is now a shift from a pandemic to an endemic with implications for how the nature of science is communicated, only a few research studies have characterised the dynamic of discourse associated with the change. This article aims to illuminate how the changes take place in terms of the linkage among different dimensions of how science works in the UK news articles when the UK is transiting from pandemic to endemic. A novel technique, epistemic network analysis (Shaffer et al., 2016) was used to quantify the connections between categories of the nature of science in these news articles. The research question below guides the present study:

*What are the connections between nature of science categories in the UK COVID-19 news articles on public health information, and how do they vary across political and temporal domains?*

## Literature review

### Science communication in news media during pandemic crisis

Science communication plays a pivotal role in fostering public trust and understanding of scientific solutions to control the spread of coronavirus, hence building up societal immunity against viral infection (Matta, 2020). Previous science communication studies focused on media conceptualisation of the pandemic crisis (Hart et al., 2020; Hubner, 2021; Ogbodo et al., 2020; Poirier et al., 2020), or employing quantitative rubric to assess the scientific quality of news on public health information (Mach et al., 2021). In the traditional model of science communication, media plays a role in filling knowledge gaps of the audience, as the audience is perceived as lacking scientific knowledge (Brossard and Lewenstein, 2009; Secko et al., 2013). However, there has been an increasing public engagement in accessing, reading and sharing COVID-19-related scientific articles (Fraser et al., 2021). Audiences became more interested in the processes of science instead of the products of science (Secko et al., 2013). To facilitate dialogue between public and scientific research (Reincke et al., 2020), it is important for the media to adopt effective strategies for communicating scientific research related to pandemic public health information.

Various strategies for effective science communication during the pandemic crisis, including clear messages, tailoring for laypersons, and delivered at appropriate platforms were documented in the literature (Hyland-Wood et al., 2021). An exclusive focus on scientific findings, masking values and failure to take into account various stakeholders’ perspectives, can undermine public trust in science (Intemann, 2023). For news media to uphold public trust and promote public engagement in science, they should avoid oversimplification or biased representation of scientific works (Abbasi, 2020; Erduran, 2020). Conditions of communicating coronavirus, namely partial reporting, downplaying threats, sensationalisation and political framing, were linked to varying public protective actions (Gollust et al., 2020). These conditions also lead to the public becoming more susceptible to misinformation and loses trust to comply with public health guidance by the government, such as getting vaccinated (Palm et al., 2021; Roozenbeek et al., 2020). In fact, the public has its own set of cognitive schemas to approach the nature of scientific knowledge and evaluate scientific evidence (Wynne, 1992). If the public is involved in dialogue in science-related debates, the public has the capability to pay attention to the technical nature of science, as well as the economic, sociological and ethical dimensions of science (Nisbet, 2016; Nisbet and Scheufele, 2009). The current study offers two innovative theoretical contributions to science communication in a public health crisis. The first contribution is to characterise how news outlets of different political domains communicate how scientific knowledge was generated. The second contribution of this study is to justify the application of an interdisciplinary framework that focuses on the balance of various dimensions of science in communicating public health information by news media.

### Communicating nature of science in COVID-19 News

To characterise media representation of various dimensions of science in communicating public health information, the nature of science framework from Erduran and Dagher (2014) was adopted. In their framework, the nature of science is viewed as comprising a social-institutional system and a cognitive-epistemic system: for the *social-institutional system*, it encompasses how scientific knowledge is shaped by the social and political dimensions, namely social certification and dissemination, social values, scientific ethos, professional activities, social organisations and interactions, financial systems and political power structures; for the *cognitive-epistemic system*, it describes scientific practices of enquiry, aims and values, methods and methodology rules, and forms of scientific knowledge. A majority of previous studies, which were conducted at the beginning of the Covid-19 pandemic, showed that media representation of public healthcare policies and measures excluively focused on only social-institutional system (Abbas, 2020; Abbasi, 2020; Chen et al., 2022; Xu et al., 2022). For example, the TV media coverage of political power structures of race was far more than that of physical outcomes during the first stage of the pandemic in the United States (Xu et al., 2022). On the other side, German news coverage of Covid-19 public healthcare policies and measures was dominated by actors from political dimensions instead of scientific experts (Leidecker-Sandmann et al., 2022). An underemphasis on the cognitive-epistemic dimension in media representation of the healthcare crisis was believed to induce fear and a lack of trust in public healthcare policies and measures (Hardy et al., 2021; Puri et al., 2020).

Despite an exclusive focus on the social-political nature of science in newspapers at the early stage COVID-19 pandemic, less is understood how media representation of the nature of scientific evidence changes while progressing into the stage of “living with the virus”. As the public becomes less sensitive to politicisation of the healthcare crisis, the media need to specify how scientific decisions were arrived at by scientists and politicians (Emanuel et al., 2022). A balanced media representation of both the cognitive-epistemic system and the social-institutional system can communicate clearly the goals and strategies for enforcing restriction, and eventually removal of restrictions during the endemic stage. This helps rebuild public health during the transition of healthcare crisis by reinforcing trust in scientific expertise, public heath institutions and belief in citizens’ actions for public interests (Makridis and Wu, 2021).

### Epistemic network analysis: a novel method in studying science communication in pandemic healthcare crisis

Quantitative content analysis of frames in news media has been a widely adopted approach to study science communication practices in news media (Evans, 2021; Ogbodo et al., 2020; Park et al., 2020; Poirier et al., 2020). These studies analysed the frequency of the presence of certain frames in the news corpus and compared the differences across topics and time using inferential statistics such as Chi-square statistics. However, such an analytical method did not consider the *connection* of scientific aspects in science communication by news media during pandemic. Epistemic network analysis (ENA) is an analytical method to characterise the frequency of connection of codes within idea units (Shaffer, 2017; Shaffer et al., 2016). This analytical method is a potential tool to characterise the frequency of connections among NOS categories. Various aspects of science, such as aims and objectives, reducing bias, and the need for science in society should be coherently addressed in science communication (Matta, 2020). It is envisaged that a news article comprises several aspects of interrelated NOS categories, which constitutes the meaning of the news article.

Characterising connections among different categories in the cognitive-epistemic system and the social-institutional system of science could be novel to science communication researchers. ENA potentially reveals differences in connections of NOS categories in news media across political and temporal domains. This enables comparison of “models” of science communication in news articles across different times of publishing and outlets with various political stances. As Secko et al. (2013, p. 64) argue, models of science communication refer to “a representation, and its associated heuristic description, of the reality of how science is communicated or how it could and/or should be communicated.”. The technique could visualise models of science communication at various timepoints of the COVID-19 pandemic. These networks of NOS connections are a metaphoric structure that satisfies concepts adopted by new media artworks (Ahmedien, 2022).

## Methodology

### Contextualised information of the UK

**Fig. 1:**
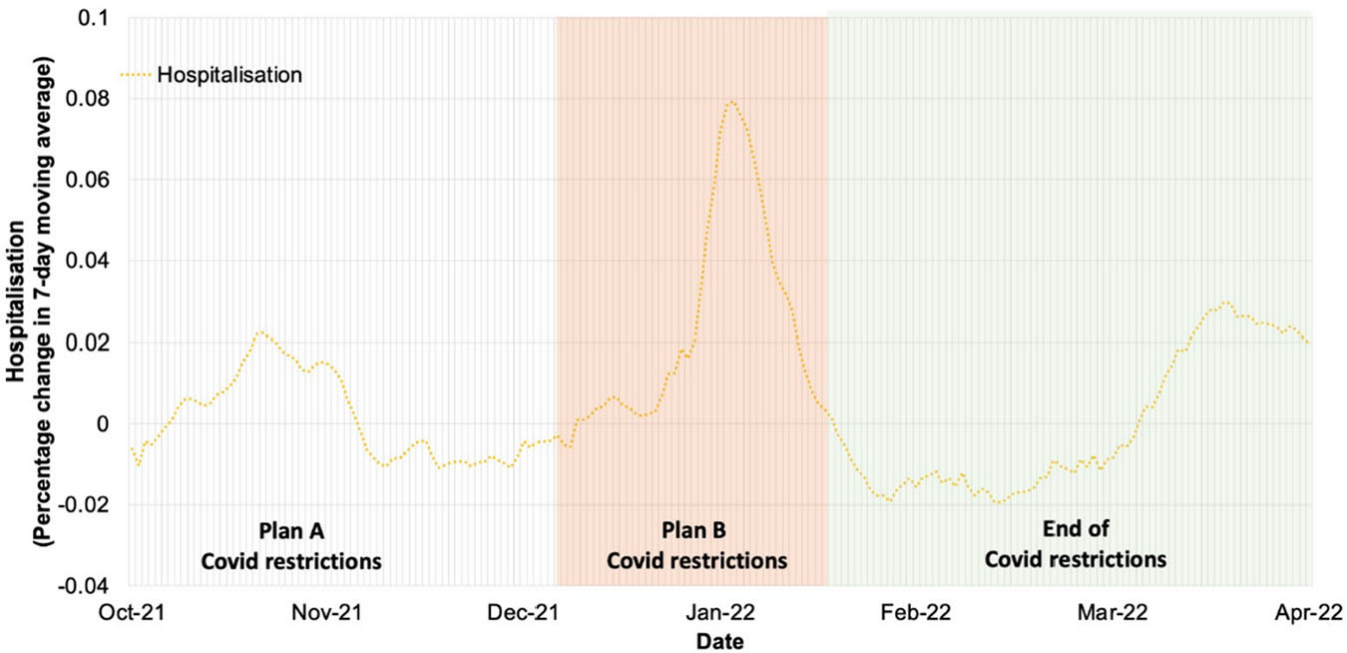
The evolution of COVID-19 hospitalisation from October to April 2022. The number of hospitalisation began to increase in Dec-21, and reached a peak in Jan-22.

Our news media analysis is contextualised with the number of COVID-19 cases, deaths, hospitalisation as well as the changes in COVID-19 policies in the UK from November 2021 to February 2022 (Fig. 1). It helps understand how representations of NOS vary regarding the shift from pandemic to endemic in terms of the changes of UK COVID-19 policies and COVID-19 situations.

The number of COVID-19 hospitalisations increases from December 2021 to January 2022 and then drops afterward (Public Health England, 2022). In January 2022, there were several news articles (see examples from Wellcome (2022) and Gallagher (2022)) claiming that the UK has the possibility to transit from the pandemic to endemic, owing to its high vaccination rate of booster dose and the reduction of hospitalisation rate of patients infected by the Omicron variant. In November, Omicron was declared a variant of concern by the World Health Organization (World Health Organization, 2021) and the first case of the Omicron variant has been identified in the UK on 27 November 2021 (Department of Health and Social Care, 2021). Our research aim is to reveal the difference in the representation of the nature of science in news articles across temporal domains.

### Inclusion and exclusion of news articles

The protocol of Mach et al. (2021) was modified to form the exclusion and inclusion protocol of our study. Print and online news articles were searched using the news data base, *Factiva*. Four news media outlets, The Guardian, The Times of London, The Telegraph and Daily Mail were selected because they represented a range of political orientations from left-wing to right-wing (Hönnige et al., 2020). The selected news publishing companies have only print and online media instead of television broadcasting, so their full articles can be systematically retrieved from Factiva (Mach et al., 2021). Although the chosen news outlets cover news in other nations in the UK, these news outlets might be more representative of the context of England. The research team has input the search terms proposed by Mach et al. (2021) in the headline and lead paragraph, namely “coronavirus”, “epidemic’, “outbreak”, “pandemic”, “SARSCoV-2” or “COVID-19”. 7756 articles with sampled dates between 1 November 2021 to 28 February 2022 were retrieved from the database. The selection of period was based on our assumption that it potentially captures three important phrases of COVID-19 situations in the UK: (1) the transition from the prevalence of the Delta variant to that of the Omicron variant, (2) the surge in the number of infections cases caused by Omicron variant, (3) the increasing number of evidence that Omicron causes less severity to human health (Torjesen, 2021) and the easing of national restrictions takes place. In this period, there were a total of 140,243 articles published in these four news outlets from the record of Factiva, which means that 5.53% of the news articles comprise the search keywords in their title and lead paragraph. All identical duplicates were removed by the search engine of Factiva.

Each news article was examined for its eligibility according to our research questions. Firstly, the selected articles should articulate public health implications, spreading or measures for controlling COVID-19 in some parts of the articles (Mach et al., 2021). By excluding news articles that are irrelevant to our research questions (i.e. activities of politicians during the COVID-19 period), the distribution of representation of the nature of science categories across temporal and political domains in communicating COVID-19-related public advice can be studied. Secondly, news articles relevant to the UK context were selected for analysis. For example, news articles centring the discussion on how mitigation efforts on the COVID-19 outbreak in Canada and India were excluded. This research study aims to identify how the UK news represents the nature of science in a way that the UK is prepared to transit from pandemic to endemic. This criterion was included because we could investigate how each article communicates the nature of scientific evidence and frame science in the news as science–society interactions and policy–science interface (Mach et al., 2021). Thirdly, articles should be original news reports. Other types of articles such as opinions, editorials, comments, and correction memos were excluded as they seldom communicate the nature of scientific evidence and are beyond the scope of the coding scheme (Mach et al., 2021). To ensure credibility in screening articles, the first two authors examined 100 news articles and refined the inclusion criteria. The remaining news articles were screened by the first author and the second author. The names, the news outlet, the date of the articles, and whether the articles fulfil the above three inclusion criteria were marked in a Google Excel spreadsheet. When each 200 news articles were screened, the authors conducted regular meetings, where the authors randomly selected a sample of news articles to check if the judgement on exclusion/inclusion of news articles was accurate. A total of 1520 articles fulfilling these three inclusion criteria were selected.

### Analysis of nature of science

To analyse and visualise the connections between categories of the nature of science, we adopt two respective coding tools. We examined the categories of the nature of science represented in each news article. The nature of science refers to how science works in both the cognitive-epistemic system and the social-institutional system (Erduran and Dagher, 2014). The framework from Erduran and Dagher (2014) was selected because their framework addresses the nature of scientific work and scientific evidence in a broad socio-cultural context. The nature of science categories for the analysis of news articles was as follows: aims and values, scientific knowledge, scientific practices, and scientific methods, social certification and dissemination, scientific ethos, social values, professional activities, social organisations and interactions, financial system and political power structures. Each article was examined in terms of the presence or absence of categories of the nature of science (see Table 1) (Bichara et al., 2021; Wu and Erduran, 2022). Each news article can include one or multiple categories of the nature of science.

**Table 1:** Definitions of nature of science categories and indicative keywords (Wu and Erduran, 2022).

Categories	Definitions	Indicative Keywords
Aims and values	Aims and values refer to a set of aims in the sense that the products of scientific activity are desired to fulfill them	Aim, value, goal, accuracy, objectivity
Methods	Methods and methodological rules refer to the variety of systematic approaches and the rules that academics and politicians use to ensure that they yield reliable knowledge	Method, scientific method, enquiry, process, hypothesis, manipulation of variables
Practices	Scientific practices refer to a diverse set of practices that are underpinned by cognitive, epistemic, and social-institutional activities by individuals and society.	Observation, experimentation, data, explanation, modelling, argumentation, classification, prediction, decision-making/action-taking based on scientific study.
Knowledge	Scientific knowledge refers to the “end product” of scientific activity that culminate in “laws, theories, models, and the collection of observational reports and experimental data”	Knowledge, scientific knowledge, formulation of knowledge, theory, law, model, shown by the study
Social certification and dissemination	Social certification and dissemination of scientific knowledge refers to the peer review process, which tends to work as “social quality control over and above the epistemic control mechanisms that include testing, evidential relations, and methodological consideration”	Peer review, validate, evaluate, certification
Scientific ethos	Scientific ethos refers to the set of norms different stakeholders in society follow in their own work and their interactions with one another	Scientific norms, ethics, bias, being sceptical, caution against bias
Social values	Social values of science refer to values such as “respect for the environment and social utility, which is broadly understood to refer to improving people’s health and quality of life as well as to contributing to economic development”	Culture, cultural, social values, society, beliefs, equity, care for elderly, freedom, respect (i.e. respect our NHS staff)
Professional activities	Professional activities refer to activities that different stakeholders, including scientists, politicians, citizens and academics, perform in order to communicate their research, such as attending professional meetings to present their findings, writing manuscripts for publications, and developing grant proposals to obtain funding	Conference, article, presentation, writing, publishing, publication
Social organisations and interactions	Social organisations refer to the role of institution and research centre in influencing scientific work.	University, research center, institution, organisation
Financial systems	Financial systems refer to the role of money on research works in science and society such as research funding.	Financial, funding, finance, economy, economical, budget, sponsor
Political power structures	Political power structures refer to how different political factors such as the role, gender, ethnicity, race and nationality in the lab and society affect scientific work.	Political power, MPs, research team, team leader, team members, researcher

### Data analysis and intercoder reliability

To ensure consistent interpretation of representations of the nature of science in news articles, three authors took part in four-month training and meeting in order to familiarise the coding framework by Erduran and Dagher (2014). First, as the framework of the nature of science from Erduran and Dagher (2014) originated from science education, we examined relevant studies in science education and identified keywords or phrases that exhibit certain categories of the nature of science (Table 1). Second, we critically examined research studies on science communication in COVID-19 news and discussed how the definitions from Erduran and Dagher (2014) could be modified to capture representations of the nature of science in the news articles. Third, the first and the second authors conducted several rounds of independent coding of the news articles, followed by negotiation and judgement on the definition and examples of codes (Mach et al., 2021). This round of discussion ensures that the inclusion of examples of key phrases and keywords can capture contextual ideas related to the era of COVID-19. For example, we considered and agreed upon including respect for medical healthcare staff in the category of “social value”.

Intercoder reliability was computed to assess the consistency of the coding tool (Cheung and Tai, 2021; O’Connor and Joffe, 2020). The first and the second authors analysed the presence of nature of science categories in 10% of the randomly selected news articles in the database. They recorded the responses through a Google form which requires information such as the name of the article, publishing date, news outlets, categories of nature of science represented. A range of Cohen’s kappa indices of 0.69–0.95 (with an average of 0.81) was obtained for analysing categories of the nature of science, indicating a good to substantial agreement (Cheung and Tai, 2021).

### Epistemic network analysis

Epistemic network analysis (ENA) is a technique that visualises the weighted connections between a relatively small number of nodes in discourse data (Shaffer and Ruis, 2017). It computes the strength of connections among between two codes based on their relative frequency of cooccurrence within a stanza that is defined by the user (Shaffer et al., 2016). The strength of connections can be reflected by both connection coefficients and the thickness of the connection lines in the networks (Shaffer, 2017). A thicker line of connections indicates a more frequent connection between two nodes, while a thinner line of connections indicates a less frequent connection between two nodes (Shaffer, 2017). The uniqueness of ENA enables researchers (1) to compare the differences in connections between nodes in various networks and (2) to convey information that is consistent with the summary statistics (Bowman et al., 2021).

Networks were created in an online software (http://app.epistemicwork.org) (Shaffer et al., 2016) to understand the connections among the nature of science categories and those between frame of science and the nature of science. For example, if a news article included a code “social certification and dissemination” and a code “social progress”, ENA constructs matrices based on the co-occurrence between them and projects codes in a high-dimensional space (Pantić et al., 2021). In ENA, singular value decomposition (SVD) reduces the dimensionality by maximum variance (Pantić et al., 2021). This way of analysis allows both visual and statistical comparison of connections of codes between networks. The two SVDs in epistemic networks, SVD1 and SVD2, account for the most variance in the discourse data (Pantić et al., 2021). In epistemic networks, the square in each network is an arithmetic mean which computes the average values in the connection weights in networks. This allows statistical tests such as two-sample *t*-test to compare differences among networks.

As stipulated in our research questions, categories of the nature of science were selected as codes. We defined ENA units as each month of news articles in each news outlet in order to compare connection structures across different political and temporal domains, therefore 16 units (4 news outlets × 4 months) were generated. the stanza size was set as one as we were interested in cumulative connections among the nature of science categories in a single news article (Shaffer et al., 2016). We then used each news ID as “conversations” (Shaffer, 2017; Cheung and Winterbottom, 2023) to model interactions among the nature of science in each stanza, that is each news article. The option of sphere normalisation was selected such that the magnitude of vectors for each unit was removed, as the networks in each newspaper outlet comprise various numbers of articles (Bowman et al., 2021). The function of normalisation enables the calculation of proportions of the occurrence of each code pair within an ENA unit, for example, a month in a newspaper outlet (Bowman et al., 2021). As the distributions of projection points were normally distributed owing to its large sample size (Swiecki et al., 2020; Zhang et al., 2022), we applied two-sample *t*-test to mathematically compare the differences between arithmetic means of different months of COVID-19 news and news outlets with different political stances reporting COVID-19 news.

## Results

### The extent of news coverage focusing on public health implications

**Fig. 2:**
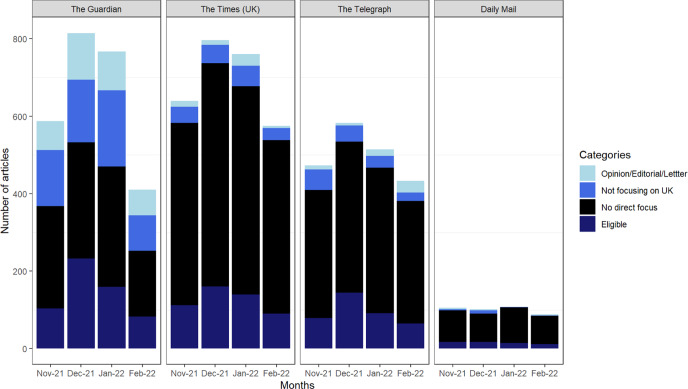
The distribution of eligible and illegible articles over different time frames across four different news outlets (The Guardian, The Times (UK), The Telegraph, Daily Mail). Ineligible articles were further differentiated into three categories, opinion/editorial/letter (light blue), not focusing on UK (royal blue), no direct focus on public health implications (black).

Four news outlets comprise various numbers of news articles focusing on public health implications related to COVID-19 from November 2021 to February 2022. Owing to a surge of Omicron variant, December 2021 recorded the highest number of news articles focusing on COVID-19-related public health implications in three news outlets, The Guardian, The Times (UK) and The Telegraph. There is not much variation in the number of eligible news articles across four months in the Daily Mail. Of 7756 news articles with keywords from Factiva returns 1520 articles (19.6 %) articles which is original news articles focusing on public health implications in the UK were included in further analysis. Among four news outlets, 578 articles in The Guardian, 502 articles in The Times (UK), 379 articles in The Telegraph and 61 articles in Daily Mail were eligible for analysis (Fig. 2) (see Appendix 1A). Further analysis on the representation of the nature of science was performed on these eligible articles.

### Distribution of nature of science categories across temporal and political domains

**Fig. 3:**
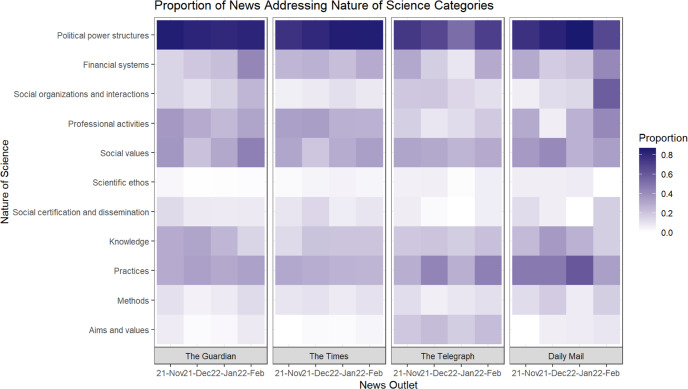
Heatmap showing the proportion of news addressing nature of science categories in different months in different news outlets. Darker purple colour indicates a higher proportion of news articles addressing a certain nature of science category, while a lighter colour indicates a lower proportion of news articles addressing a certain nature of science category.

Before conducting an epistemic network analysis of the connections among the nature of science categories in various news outlets, we highlight the variation in the proportion of news articles addressing a certain nature of science category across temporal and political domains. As the number of articles is different across months in each news outlet, we normalised the results by dividing the number of news articles addressing a category of nature of science by the total number of eligible news articles in each month of a news outlet. A heatmap (Fig. 3) shows variations across different time periods in four selected news outlets, arranging from left-winged news outlets (The Guardian) to right-winged news outlets (Daily Mail) (refer to frequency of representation of nature of science and proportion of news addressing nature of science categories in Appendices 1B and 1C).

News articles extensively focuses on the category of “political power structures” across temporal and political domains (The Guardian: 81.8%; The Times: 81.1%; The Telegraph: 61.4%; Daily Mail: 78.7%). As indicated in the heat map in Fig. 3, “political power structures” has the darkest colour among all eleven nature of science categories. This shows that the role of political factors such as government and MPs was commonly addressed in the news that communicates public health implications related to COVID-19. Compared to other months, “financial systems’ are mostly emphasised in news in February 2022 in three news outlets (The Guardian: 42.2%; The Times: 28.9%; Daily Mail: 41.7%). As the UK government positioned it as a country in an endemic state in February 2022, the news articles mostly focus on the economic advantages of the British reopening policies.

Two categories in the cognitive-epistemic system and two categories in the social-institutional system are downplayed across temporal and political domains. For the cognitive-epistemic system, less than 10% of the news articles address “aims and values” across all publishing months in three news outlets (The Guardian: 3.63%; The Times: 1.60%; Daily Mail: 4.92%). The references to the goal, accuracy, and objectivity of science in the news articles are scarce across these three news outlets. Moreover, in each month of the news, less than 15% of the news articles articulate “methods” (The Guardian: 7.44%; The Times: 8.96%; The Telegraph: 7.86%). Scientific methods such as observational studies in epidemiology and trials of COVID-19 vaccines are not commonly mentioned in these three news outlets. For the social-institutional system, less than 7% of the news articles represent “scientific ethos” across all publishing months of all news outlets (The Guardian: 1.04%; The Times: 3.39%; The Telegraph: 4.18%; Daily Mail: 4.92%). The scientific norms which different stakeholders followed were underrepresented in COVID-19 news, though they are important in guiding the scientific discovery of COVID-19 medical treatments and vaccines. In addition, <17% of the news articles address “social certification and dissemination” across all publishing months in all news outlets (The Guardian: 8.13%; The Times: 9.76%; The Telegraph: 2.93%; Daily Mail: 8.20%).

### Means, connection coefficients and mathematical comparisons of epistemic networks: An example from the Guardian news

**Fig. 4:**
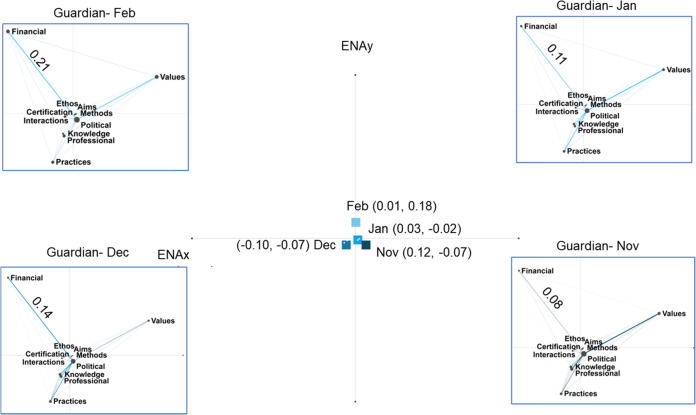
Epistemic networks of news articles addressing nature of science categories in different months of The Guardian news outlets. The locus of the arithmetic network means in each publishing month of The Guardian news are indicated by squares.

In terms of ENA networks, the nodes which are projected into the ENA space correspond to each category of the nature of science (refer to Table 1). Edges in between the nodes correspond to the frequency of weighted connections between one category of nature of science to another (Shaffer, 2017). A thicker edge indicates a more frequent connection which can be reflected numerically by connection coefficients. As shown in the example of the Guardian news across different months (Fig. 4), the connection between “financial systems” and “political power structures” is the strongest in February 2022, indicated by the connection coefficient of 0.21.

The horizontal axis of the ENA space (ENAx) depicts the representation of nature of science in news articles as the right side with social values, and the left side with financial systems and practices. The vertical axis of the ENA space (ENAy) depicts the representation of the nature of science in news articles as the upper side with financial systems and social values, and the downside with knowledge, professional activities, scientific knowledge and scientific practices. For each ENA network, there is an arithmetic mean of edge weights of each network, as indicated by the squares in Fig. 4. A series of *t*-tests were conducted to compare the *x*-locus and *y*-locus of means of a pair of ENA networks. We illustrate a mathematical example of comparing the mean loci of two ENA networks using the Guardian news published in November 2021 (Guardian-Nov) and December 2021 (Guardian-Dec). In the Guardian news, the locus of the mean of Guardian-Nov news network is *x* = 0.12, *y* = −0.07; while the locus of the mean position of Guardian-Dec news network is *x* = −0.10, *y* = −0.07. Compared to the mean locus of Guardian-Dec network, the mean locus of Guardian-Nov ENA network is statistically significantly different at the *α* = 0.05 level along *x*-axis (*t*(177.43) = 3.23, *p* = 0.00, Cohen’s *d* = 0.40). However, the mean locus of Guardian-Nov ENA network is not statistically significantly different at the *α* = 0.05 level from that of Guardian-Dec network along *y*-axis (*t*(227.14)= −0.01, *p* = 0.99, Cohen’s *d* = 0.00). As the connection between the financial system and political power is stronger in the ENA network of Guardian-Dec than that in Guardian-Nov, the mean projection points of Guardian-Dec shifts to the left along *x*-axis.

### Connection coefficients of epistemic networks of news across temporal and political domains

**Fig. 5:**
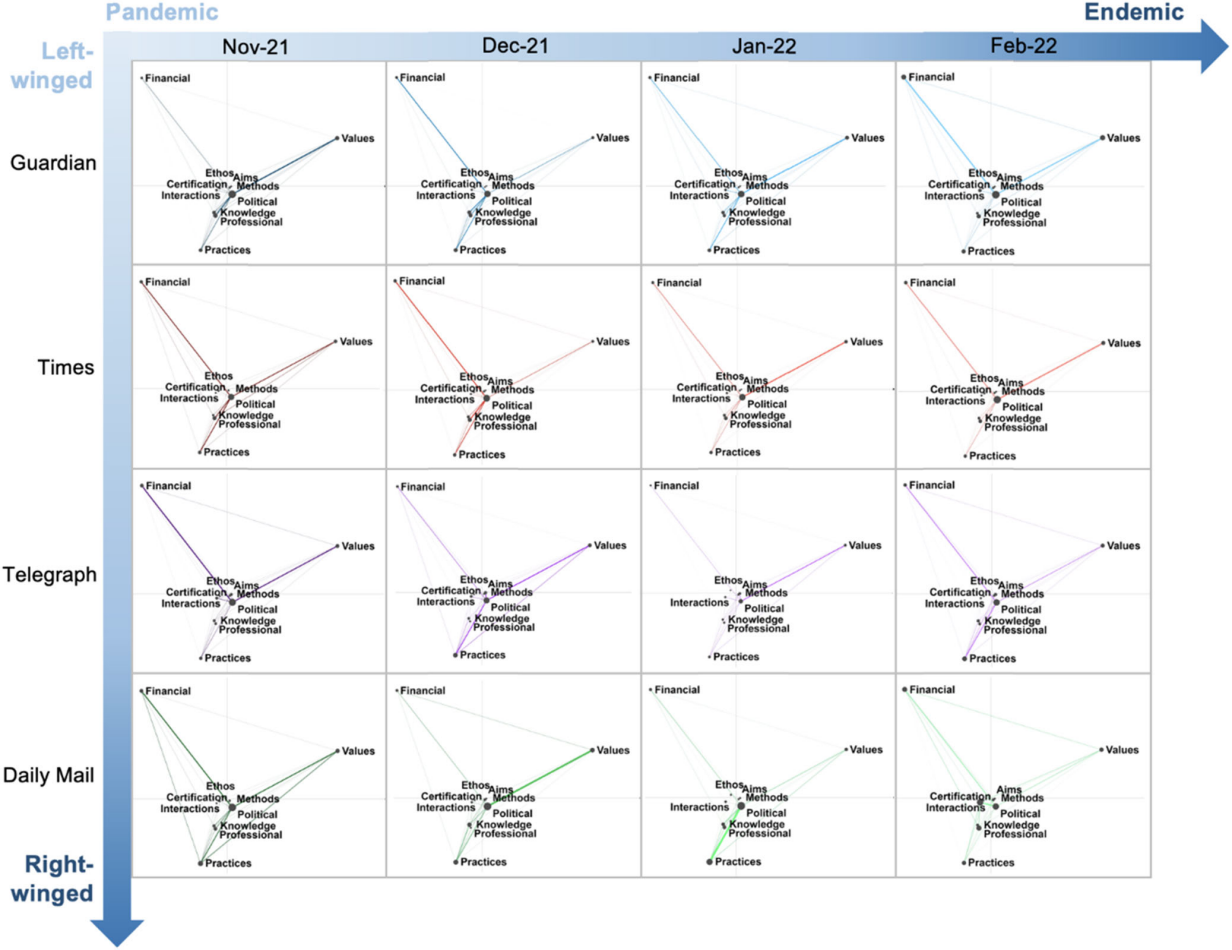
Epistemic networks showing connections of nature of science categories across time frames across four different news outlets (The Guardian, The Times (UK), The Telegraph, Daily Mail).

**Fig. 6:**
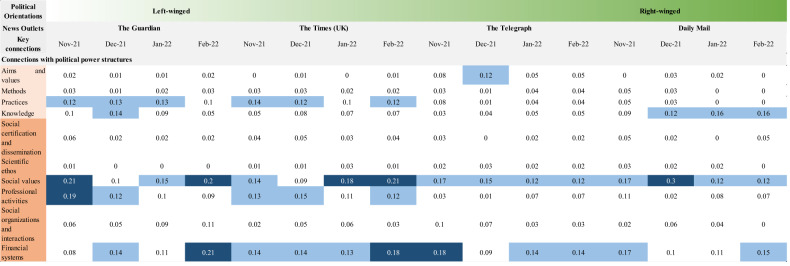
Connection coefficients (CCs) between political power structures and other nature of science categories in news articles across political and temporal domains (dark blue: CCs ≥ top 1% (0.18); blue: CCs ≥ top 5% (0.12)).

Sixteen epistemic networks were generated across four months of news articles in four news outlets (Fig. 5), and the connection coefficients (CCs) among nature of science categories (Appendix 2). Top 1% (≥0.18) and top 5% of CCs (≥0.12) were identified among all possible combinations of connections. All ENA networks showed that “political power structures” is centrally linked to other categories of the nature of science. This is also commensurate with the fact that the top 5% of CCs (0.12) are mostly distributed across connections between “political power structures” and other categories of nature of science. Therefore, CCs of “political power structures” with other nature of science categories were examined closely, which is shown in Fig. 6.

For similarities in representations of nature of science across political domains of news outlets, most of the CCs between “political power structures” and “social values” (CCs range: 0.09–0.3), as well as that between “political power structures” and “financial systems’ (CCs range: 0.08–0.21) are at top 5%. For differences in representations of nature of science across political domains of news outlets, the linkage between “political power structures” and “practices”, as well as that between “political power structures” and “professional activities”, were stronger in left-populated and centralist news outlets than in right-populated news outlets. The CCs between “political power structures” and “practices” in news published by left-populated and centralist news outlets are mostly top 5% (The Guardian: 0.1–0.13; The Times (UK): 0.1–0.14), while the CCs between “political power structures” and “practices” in news published by right-populated news outlets are lower than the top 5% (The Telegraph: 0.01–0.08; The Times (UK): 0–0.05). In addition, the CCs between “political power structures” and “professional activities” in news published by left-populated and centralist news outlets are mostly top 5% (The Guardian: 0.09–0.19; The Times (UK): 0.11–0.15), while the CCs between “political power structures” and “professional activities” in news published by right-populated news outlets are lower than the top 5% (The Telegraph: 0.01–0.07; The Times (UK): 0.02–0.11).

Regarding the patterns across temporal domains of news articles, there is not much variation in the representation of the nature of science across different times of publishing. However, in left-populated and centralist news outlets, The Guardian and The Times (UK), the connection between “political power structures” and “financial systems” (CC in The Guardian: 0.21; CC in The Times (UK): 0.18), and that between “political power structures” and “social values” (CC in The Guardian: 0.2; CC in The Times (UK): 0.21), are the strongest in February 2022. It might be because these left-populated and centralist news outlets disseminate the relation between political factors and economic factors and between political factors and the social values of “living with the virus” in repositioning the country to an endemic phrase. Another important finding is that representation of the relations between “political power structures” and categories in the cognitive-epistemic system is found in some news outlets in December 2021. For example, the CC (0.14) between “political power structures” and “knowledge” is the highest in December 2021 within the Guardian news articles; the CC (0.12) between “political power structures” and “aims and values” is the highest in December 2021 within the Telegraph news articles. This might be accounted for by the fact that the rise in the omicron variant triggers more news discussion on the relations between the role of political factors and cognitive-epistemic factors.

### Two sample *t*-tests to compare means of ENA networks across temporal and political domains

**Fig. 7:**
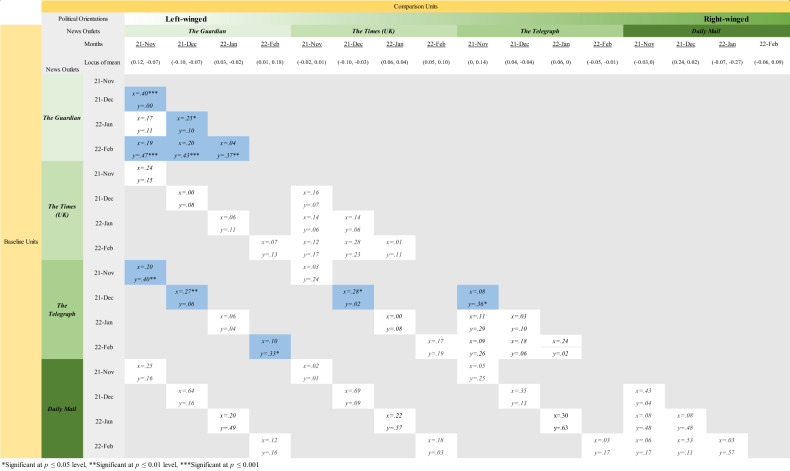
Effect sizes of differences in positions of means of the epistemic networks in terms of its *x*-axis and *y*-axis positions, as computed by two-sample *t*-tests (blue boxes indicate a significant change in position of means).

To mathematically compare the arithmetic means of networks across temporal and political domains, two sample *t-*tests were conducted to compare a pair of ENA networks. Comparisons of networks of representation of the nature of science were carried out across different months of publishing within the same news outlets, as well as those across different news outlets in the same month. Effect sizes of the loci of the network mean along the *x*-axis and *y*-axis, as well as their significant levels at the *α* = 0.05 level were computed (Fig. 7). The figure reveals that within the Guardian news, all networks differ significantly across temporal domains apart from comparing that of Guardian-Nov and that of Guardian-Jan. For example, the news network in Guardian-Nov (mean: *x* = 0.12, *y* = −0.07) has a statistically significant mean compared to Guardian-Dec (means: *x* = −0.10, *y* = −0.07) along the *x*-axis (*t*(177.43) = 3.23, *p* = 0.00, Cohen’s *d* = 0.40). On the other hand, the news network in Guardian-Nov (mean: *x* = 0.12, *y* = −0.07) has a statistically significant mean compared to Guardian-Feb (means: *x* = 0.01, *y* = 0.18) along the *y*-axis (*t*(168.26)= −3.15, *p* = 0.00, Cohen’s *d* = 0.47).

Another trend observed is that the means of networks of nature of science represented in November 2021, December 2021 and February 2022 news published by The Guardian significantly differ from those published by The Telegraph. The news network in Guardian-Nov (mean: *x* = 0.12, *y* = −0.07) has a statistically significant mean compared to Telegraph-Nov (mean: *x* = 0, *y* = 0.14) along the *y*-axis (*t*(159.98) = −2.63, *p* = 0.01, Cohen’s *d* = 0.40); the mean of a news network in Guardian-Dec (means: *x* = −0.10, *y* = −0.07) is statistically significantly different from Telegraph-Dec (mean: *x* = 0.04, *y* = −0.04) along the *x*-axis (*t*(289.12) = −2.52, *p* = 0.01, Cohen’s *d* = 0.27); the mean of a news network in Guardian-Feb (means: *x* = 0.01, *y* = 0.18) is statistically significantly different from that in Telegraph-Feb (means: *x* = −0.05, *y* = −0.01) along the *x*-axis (*t*(136.90) = 1.99, *p* = 0.05, Cohen’s *d* = 0.33).

## Discussion

The current study informs science communication literature by demonstrating the use of NOS theoretical tools to analyse how news media engaged the public in the processes of science, as well as using ENA to analyse connections between scientific aspects in news media. During the COVID-19 pandemic, the public had their own epistemological beliefs about science and was proactive in understanding how scientific knowledge is formed within wider socio-institutional contexts (Fraser et al., 2021; Intemann, 2023; Matta, 2020). A balanced representation of cognitive-epistemic aspects and social-institutional aspects of science is necessary for communicating unbiased information (Abbasi, 2020; Hart et al., 2020; Hartley and Vu, 2020). Although previous studies used coding tools such as scientific quality, sensationalism, framing and actors of science to examine the scientific representations of COVID-19 public health information (Leidecker-Sandmann et al., 2022; Mach et al., 2021; Ogbodo et al., 2020), we demonstrated the use of an interdisciplinary framework, nature of science (Erduran and Dagher, 2014), to explore the extent to which news media in UK reported various aspects of scientific works. By using this theoretical framework, the findings illustrate that aims and values and scientific methods are downplayed in news communicating the cognitive-epistemic nature of science; scientific ethos and social certification and dissemination are also downplayed by news outlets communicating the social-institutional nature of science (Fig. 3). For the cognitive-epistemic aspects of science, the goal of scientists and the methodologies scientists use were less represented in UK COVID-19 news; while for the social-institutional aspects of science, the ethical norms of guiding vaccine trials, and whether the findings on COVID-19 vaccines were peer-reviewed were underrepresented in news articles. Although news media play a central role in shaping public perceptions of science in public health crises, science is often communicated as a final product, instead of engaging the public in the process of how science works (Erduran, 2020). Ignoring ‘how’ and ‘why’ of scientific investigations are carried out at the expense of ‘what’ these investigations concluded potentially can mislead or even misinform the public when the public cannot understand the justifications, the tools and the processes through which scientific knowledge is generated.

Apart from characterising the proportion of news articles addressing each category of nature of science, this study applied a novel discourse analysis technique, epistemic network analysis, to measure how frequently two nature of science categories are connected to each other. By visualising the connections between categories (Shaffer, 2017) in news coverage across political and temporal domains, similar to the findings reported by previous studies (Abbas, 2020; Iwendi et al., 2022), our findings indicate that the political dimension of COVID-19 news is often addressed together with financial aspects. In addition, our study also reveals that social values are often connected with political dimensions. It might be attributed to social values such as awareness of civility and respect for the National Health Service and social protection for vulnerable groups, which were often reported in news media together with the government’s enforcement of Covid-19-related policies.

Despite an overemphasis on political dimensions by all news outlets, practices of science and professional activities were more often addressed together with political dimensions in the left-populated and centralist news outlets. This implies that activities specific to how scientific investigation is conducted by scientists, as well as ways to communicate scientific evidence behind these COVID-19 healthcare policies and measures, were downplayed in the right-populated news outlets. Previous research has indicated that the scientific quality of left-populated newspapers reporting COVID-19 policies and measures was higher (e.g., Mach et al., 2021). However, our findings point to the reason why the scientific quality of left-populated news outlets was higher because they tend to report the professional activities and practices of science activities in public healthcare crises. Incommensurate with the public engagement model by Secko et al. (2013), members of the public, or even news reporters themselves, can be trained to use this NOS tool to discern bias in media reporting public health information. This tool is easily understood because it has been demonstrated that the public with a range of age groups can apply this tool (Akbayrak and Kaya, 2020; Goren and Kaya, 2022). Thus, it can become a shared language between experts and the public to fact-check biased information. For instance, the public can detect the lack of scientific methods and scientific practices that derive public health information in some news media, becoming more aware of the partiality of information communicated. News media can also allow the public to express their appeal for finding information about scientific methods and practices, offering two-way communication (Matta, 2020). Hence, this helps mitigate adverse effects that emerged from biased and sensational information in future pandemics like COVID-19 or other public health emergencies.

Importantly, when news outlets report the exit of the COVID-19 pandemic in Feb 2022, social values and financial systems were addressed frequently together with political dimensions in left-populated and centralist news outlets (*The Guardian* and *The Times (UK)*). This contrasted with right-populated news outlets (*The Telegraph and Daily Mail*) which did not significantly vary across different times in terms of connections between social-institutional categories of the nature of science. This difference also suggests that left-populated outlets and centralists inform the public of “new normal” in the endemic phase by specifically highlighting the economic consequences and social value of improving the quality of life of citizens. Previous studies have reported that pandemic news coverage induced negative sentiment (Aslam et al., 2020; Iwendi et al., 2022) and was dominated by political actors (Leidecker-Sandmann et al., 2022; Xu et al., 2022). The characterisation of connections between the nature of science categories provides a more objective and sophisticated indicator of how two aspects of scientific works were addressed in reporting public healthcare crises. It reveals which specific characteristic of scientific knowledge was downplayed in a temporal or political domain.

Variation in epistemic networks of the nature of science across temporal domains was found in the left-populated news outlet (*The Guardian*) (see Fig. 7). Such variation raises a question for science communication researchers on whether news media should consistently address the same connections of nature of science at different stages of pandemic. From our results, there is a strong connection between scientific knowledge and political power structures, as well as scientific practices and political power structures in December 2021 (Fig. 5), a period when the number of Omicron cases was rising. Scientific knowledge and practices were two nature of science categories that are positively related to whether the public abides by social distancing policies (H-Y Chan et al., 2023). These connections faded along with the British government’s narrative of “exiting pandemic” and abolishment of social distancing policies starting from January 2022 to February 2022. On one side, such strategic variation in science communication by news media might have helped mobilise public efforts in following social distancing policies; while on the other hand, this way of science communication might deprive opportunities for public to understand the cognitive-epistemic aspects of science in public health information.

The limitation of this study is that four months of news articles published across four news outlets were studied. Compare to the random sampling approach by Mach et al. (2021) on these four news outlets, we sampled and selected all news articles covering scientific information regarding public health and policy at the onset of Omicron waves. A larger sample size counterbalanced the relatively short period of time studied. Future research can also compare NOS representations in news media in different COVID-19 waves. Another possible research direction is that researchers can how the public reacts to news articles with the presence of different NOS categories. These research studies can provide information on which NOS categories communicated in news media promote or undermine public trust and support in measures during public health emergencies. As demonstrated in our previous study, scientific methods and practices were related to the public’s social distancing behaviour (H-Y Chan et al., 2023). It is envisaged that experimental studies would look further into this, and provide an alternative line of research aside from studying the effects of framing on public support and trust in healthcare policies (e.g., Carreras et al., 2021; Palm et al., 2021).

## Conclusion

The application of the nature of the science framework (Erduran and Dagher, 2014; a systematic review study by Cheung, Erduran (2022)), coupled with epistemic network analysis, leads us to a better understanding of which specific aspects of scientific work are downplayed or emphasized in the COVID-19 related articles in the UK newspapers. In this study, we have carried out a content analysis of four news outlets (*The Guardian*, *The Times (UK)*, *The Telegraph* and *Daily Mail*), and compared the representation of the nature of science across political and temporal domains. News media coverage of the cognitive-epistemic nature of science was limited. More importantly, news articles often engaged in reporting several aspects of socio-political dimensions of science. A balanced representation of these nature of science categories might reinforce public trust in the government’s decision in communicating “the new normal” (Emanuel et al., 2022) or “living with the virus” (Gallagher, 2022). The findings of this study have shown that despite the politicisation of science by all news outlets, these news outlets politicise science to different extents as they linked up with other dimensions of scientific knowledge in communicating healthcare crises. In disseminating changes in guidance for healthcare stakeholders (Laing, 2011), there is a need for addressing cognitive-epistemic aspects behind guidance, such as the scientific method that derives the government’s advice (Brusselaers et al., 2022). The framework used in this study provides a potential tool for a balanced representation of scientific works in science communication during a healthcare crisis. Given the paper focuses on the analysis of articles in the news media, the study can only have a limited stake in countering the pandemic. However, the media can still be a powerful tool for society in dealing with future pandemics and epidemics by educating the public about the scientific underpinnings of health crises. Future research could potentially carry out further studies that provide the possibility of comparing the UK-based outcomes with news media from other countries to illustrate the international dimensions of the findings presented by the paper.

## Supplementary information

Appendix 1

Appendix 2

## Data Availability

The datasets generated during and/or analysed during the current study are available from the corresponding author on reasonable request. The numerical data reported in this study are attached as supplementary information.
