# Supplementary material for: Communicating science in the COVID-19 news in the UK during Omicron waves: exploring representations of nature of science with epistemic network analysis
Source: Humanit Soc Sci Commun. 2023 Jun 5;10(1):282. doi: 10.1057/s41599-023-01771-2 (PMC10240474; doi:10.1057/s41599-023-01771-2)

**Appendix 1A**. The distribution of eligible and illegible articles over different time frames across four different news outlets


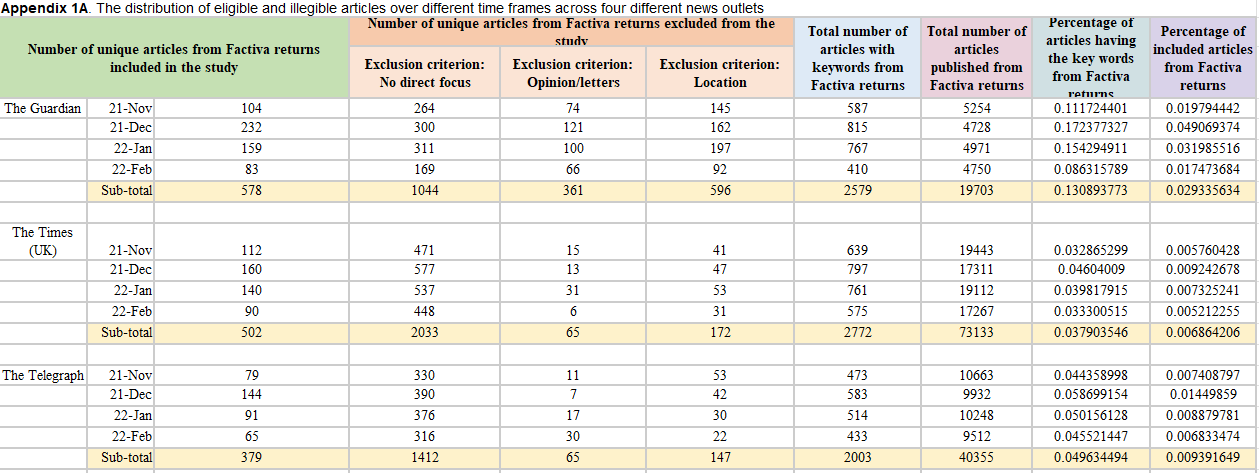


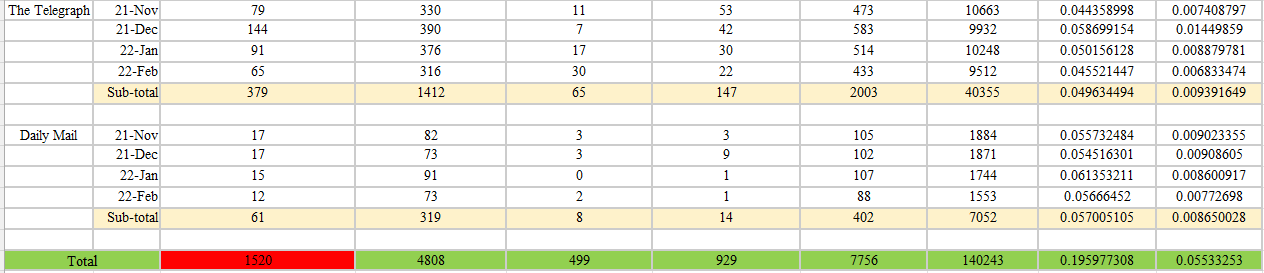


**Appendix 1B**. Frequency of representation of nature of science across different political and temporal domains


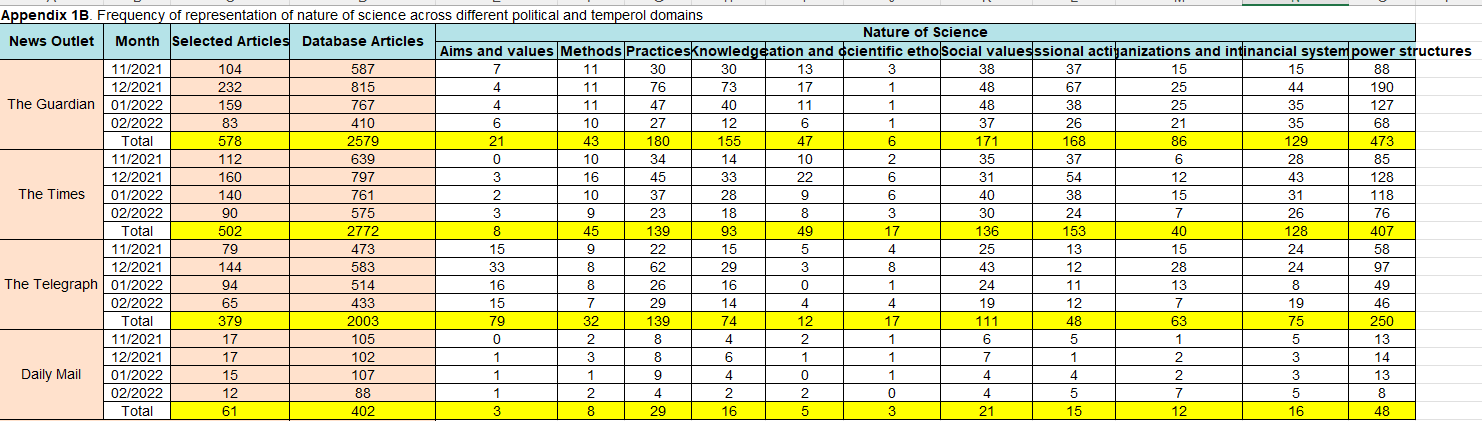


**Appendix 1C**. Proportion of news addressing nature of science categories across different political and temporal domains


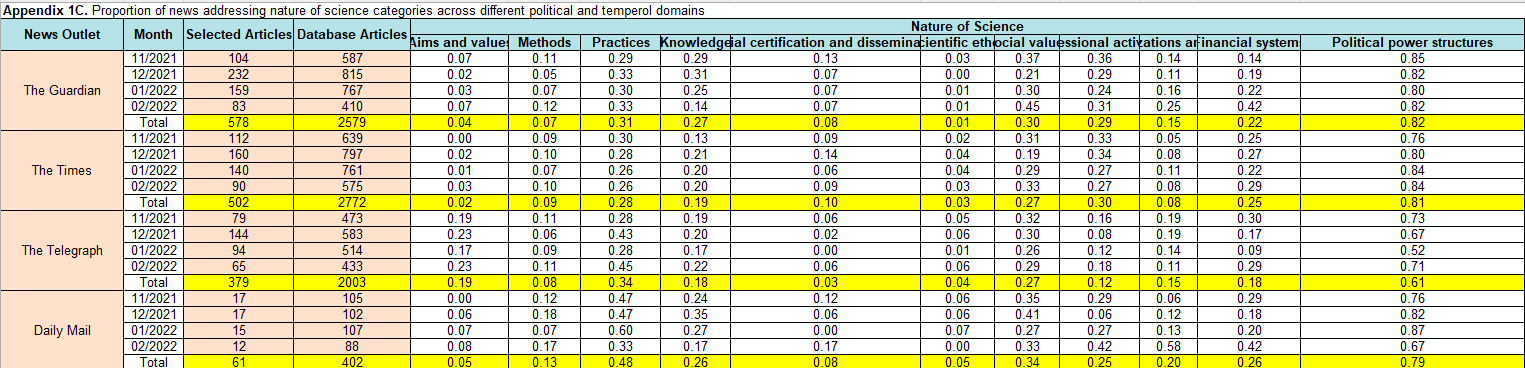

Supplement: Supplementary file 1 — Appendix 1 [file 41599_2023_1771_MOESM1_ESM.docx]
