# Supplementary figures and images for: Communicating science in the COVID-19 news in the UK during Omicron waves: exploring representations of nature of science with epistemic network analysis
Source: Humanit Soc Sci Commun. 2023 Jun 5;10(1):282. doi: 10.1057/s41599-023-01771-2 (PMC10240474; doi:10.1057/s41599-023-01771-2)

**Appendix 2.** Connection coefficients (CCs) among nature of science categories


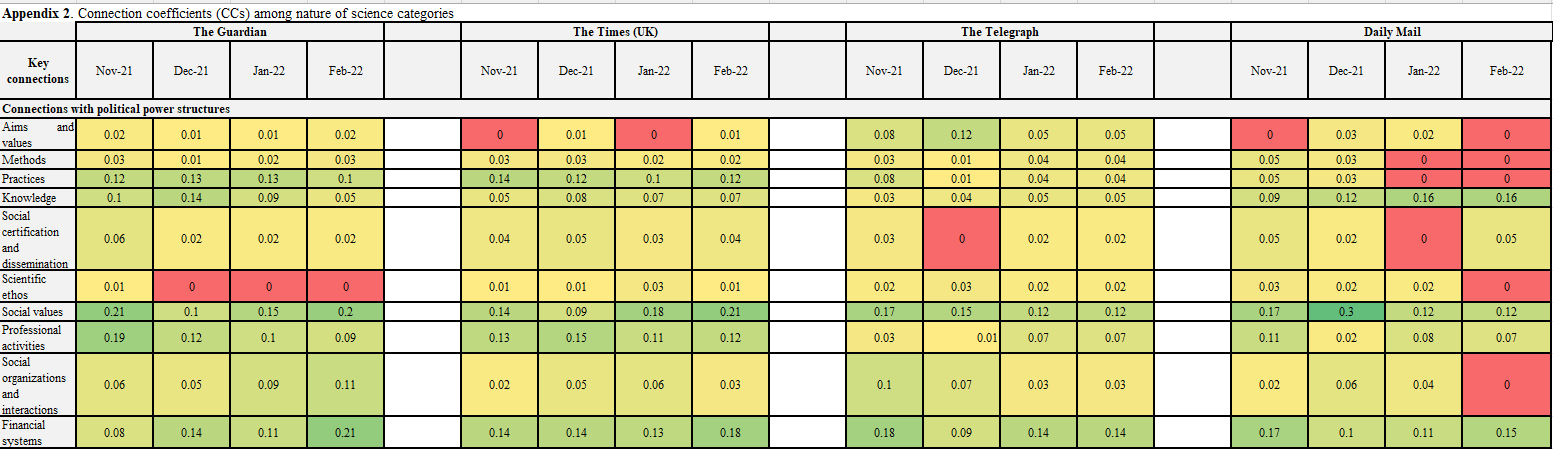


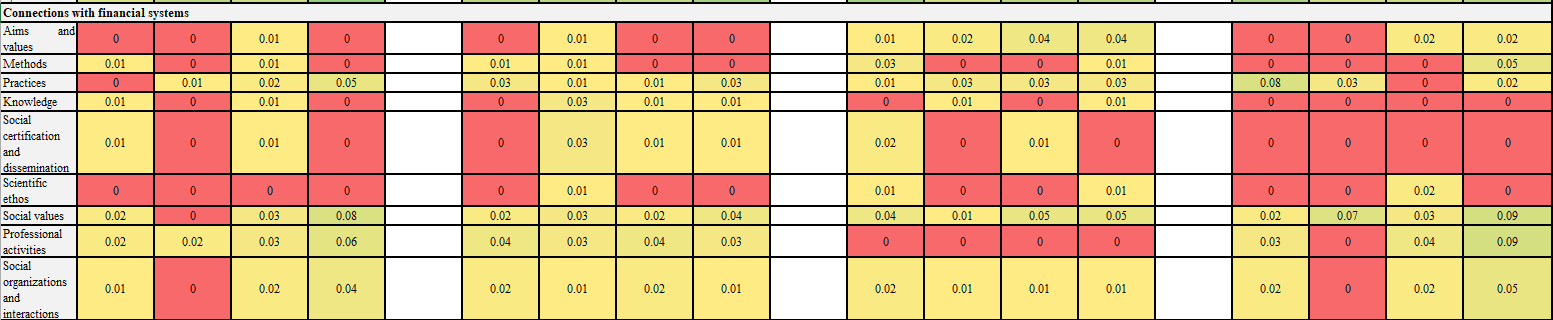


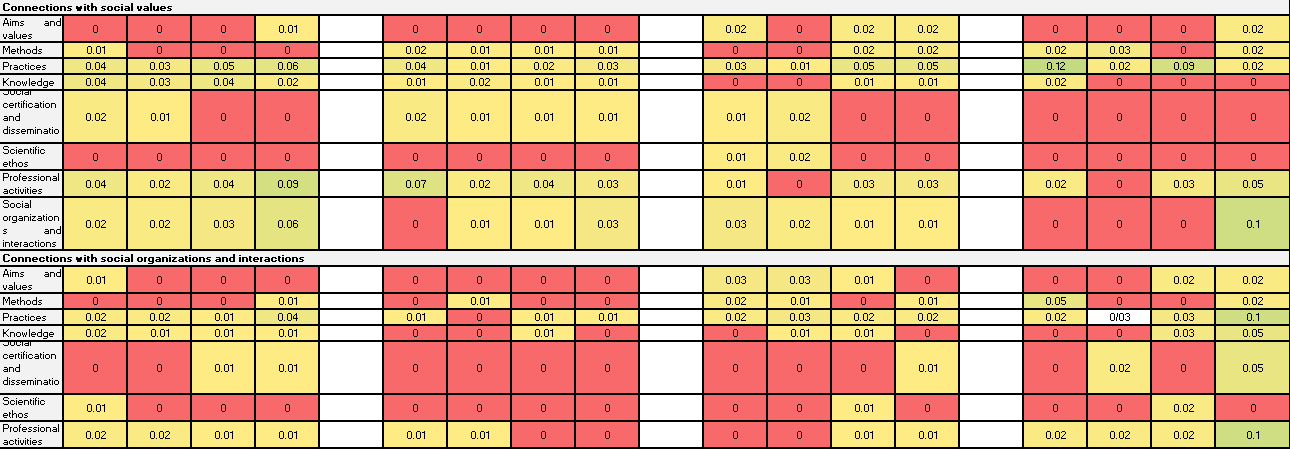


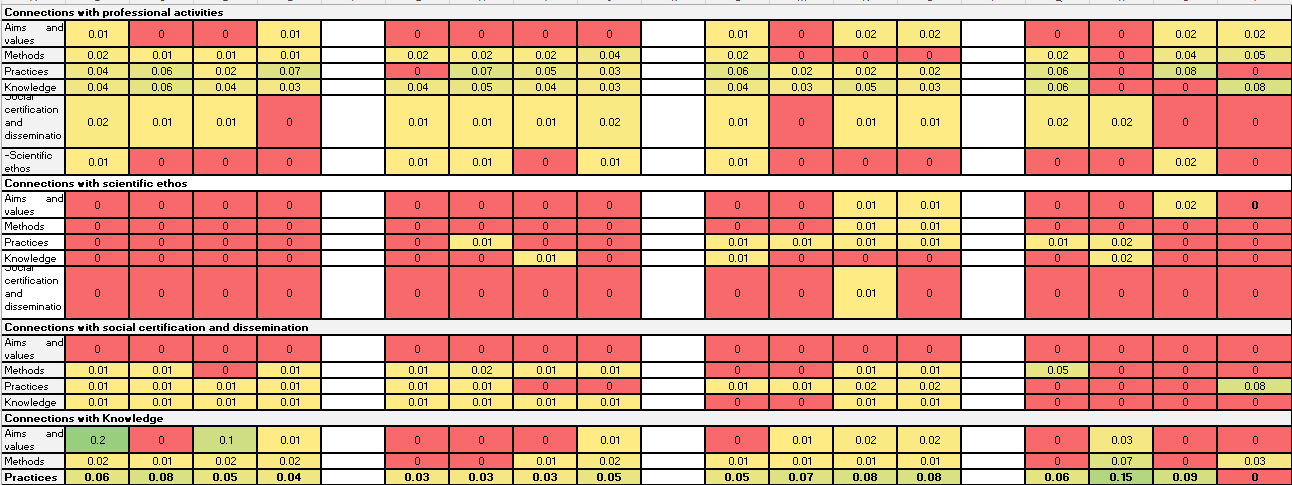


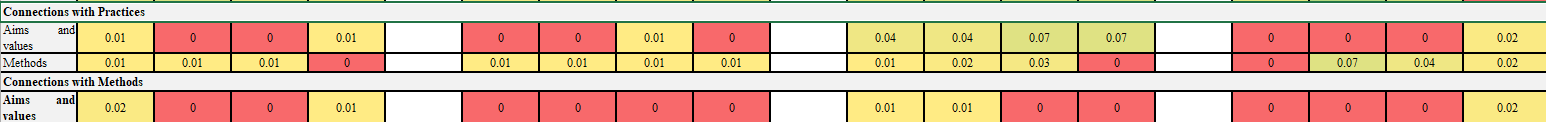

Supplement: Supplementary file 2 — Appendix 2 [file 41599_2023_1771_MOESM2_ESM.docx]
